# Self-management interventions for adult haemodialysis patients: a scoping review of randomized controlled trials

**DOI:** 10.1186/s12882-025-04229-6

**Published:** 2025-06-13

**Authors:** Nina Katharina Friedrich, Nico Schmitt, Helena Hruby, Christiane Kugler

**Affiliations:** Faculty of Medicine, Institute of Nursing Science, University of Freiburg, Breisacher Str. 153, 79110 Freiburg, Germany

**Keywords:** Dialysis, End-stage kidney disease, Self-management, Randomized controlled trials, Scoping review

## Abstract

**Background:**

Effective self-management is crucial for individuals undergoing haemodialysis to prevent complications, which can lead to morbidity and mortality. However, among this population self-management behaviours are often inadequate. To improve patient outcomes interventions that promote and enhance self-management behaviours are essential.

**Objective:**

This study aimed to provide an overview of the current body of evidence from randomized controlled trials (RCTs) on self-management interventions for haemodialysis patients. First, we aimed to present the outcomes investigated, their corresponding measurement tools and the significant results of each study. Second, we examined the presence of various self-management components and activities within the interventions. We aimed to identify new, underrepresented, and absent self-management components and activities found in the interventions.

**Methods:**

Following the scoping review process, a systematic literature search was conducted across six databases (MedLine All, Emcare, CINAHL, PsycINFO, Web of Science, Cochrane) to identify relevant studies. The search focused on RCTs involving adult haemodialysis patients. The review utilized the Preferred Reporting Items for Systematic Reviews and Meta-Analyses Statements for Scoping Reviews (PRISMA-ScR) and the Joanna Briggs Institute (JBI) approach. The included studies were required to address either self-management theories or self-management support interventions. Data were synthesized using a tabular format. The findings were analysed using content analysis based on the self-management concept.

**Results:**

Overall, fourteen articles from eight countries were included. The findings illustrate the broad thematic scope of self-management interventions. Most frequent intervention outcomes were ‘quality of life’, ‘self-management’ and ‘self-efficacy’. The most used instrument was ‘Strategies used by people to promote health’ for measuring self-care self-efficacy. All authors of the included studies reported significant results of the intervention. The content analysis identified commonly employed self-management components and activities for haemodialysis patients, such as ‘emotion regulation’, ‘medication adherence’, and ‘diet management’. It revealed underrepresented (infection control), absent (smoking cessation), and new (stress management) self-management dimensions.

**Conclusion:**

This innovative scoping review represents the first comprehensive overview of self-management intervention outcomes, their measurements, and significant results while simultaneously highlighting the complex self-management components and activities that haemodialysis patients must navigate on a daily basis. The identified gaps and opportunities underscore important areas for future intervention development.

**Clinical trial number:**

Not applicable.

**Supplementary Information:**

The online version contains supplementary material available at 10.1186/s12882-025-04229-6.

## Introduction

Approximately eight million people, 0.1% of the world’s population, are estimated to have kidney failure [1, 2]. Kidney failure, formerly named end-stage kidney disease, means that kidney function has declined so severely that renal replacement therapy is necessary to sustain life [1, 3]. Based on a global median prevalence of 397 per million people, approximately 3.18 million individuals worldwide are estimated to be undergoing chronic dialysis, the most commonly used form of renal replacement therapy [1]. Haemodialysis, the most frequent form of dialysis, is a medical procedure that filters waste, toxins, excess fluids, and electrolytes from the blood by diverting it via a vascular access through a machine with a specialized filter, thereby partially replicating kidney function [3].

When starting dialysis, patients encounter new challenges, requiring to restructure their daily routines and acquire new skills [4]. During conservative chronic kidney disease treatment without renal replacement therapy, patients must manage a comprehensive therapeutic regimen including diet and fluid restrictions. After transitioning to dialysis, they must manage additional aspects such as vascular access care and strict dialysis schedule adherence to prevent complications [5]. Patients describe haemodialysis as a demanding treatment regimen, typically requiring three or more sessions per week or more, each lasting four to five hours [1]. While essential for survival, this intensive schedule affects patients’ daily lives including financial status, social roles and perceived quality of life [6–8] and is associated with higher morbidity and mortality rates [9, 10]. Patients can experience side effects and symptoms such as fatigue (67-78%), bone and joint pain (70.9%) [11, 12], anxiety (28%) and/or sadness or depressive symptoms (30-49%) [9, 13].

Adhering to the complex therapeutic regimen, however, is particularly challenging for haemodialysis patients. Recent studies have described patients experiencing difficulties in implementing lifestyle changes [14], which can lead to inadequate self-management [15]. Self-management is a comprehensive concept that encompasses all activities necessary to manage disease and its treatment in order to achieve, maintain or promote optimal health [14, 15] 16). In populations with chronic illnesses, self-management refers to patients’ ongoing active participation in their own care as well as their ability to independently manage and cope with the consequences of their health conditions [16, 17].

In haemodialysis patients, evidence suggest that effective self-management not only alleviates associated burdens and reduces symptoms, but also enhances overall health-related quality of life [16, 17]. Schäfer-Keller [18] described a self-management model for renal replacement therapy patients that is divided in three components: ‘managing the medical regimen’, ‘managing emotions’ and ‘managing (new) life roles’. Each component includes different self-management activities [15, 18].

To improve patient self-management, it is essential to develop interventions aimed at supporting and guiding patients throughout this process. On the one hand, several self-management interventions have been developed with focus on haemodialysis patients acknowledging their specific situation, each focusing on a broad scope of different outcomes [16, 19]. On the other hand, these interventions encompass various self-management activities such as managing therapeutic regimens, maintaining emotional well-being, or adjusting to new life roles [20–22]. However, there is a lack of a comprehensive overview that explicitly addresses both, the variety of self-management interventions for haemodialysis patients and the diverse range of self-management activities.

To the best of our knowledge, no review has systematically outlined self-management interventions for haemodialysis patients, which presents outcomes and their measurement instruments as well as the significant results of applied interventions. Furthermore, no review has summarized the different self-management components and activities as well as their gaps related to haemodialysis patients. An overview of self-management interventions for haemodialysis patients could serve as a valuable foundation for guiding the development of future self-management interventions. Therefore, we focused on randomized controlled trials (RCTs), as they provide the highest level of evidence for assessing intervention effectiveness and thereby offer critical insights for the development and implementation of self-management programs in clinical practice.

The aim of this scoping review was to summarize the current evidence on self-management interventions for haemodialysis patients, to present their outcome and measurement instruments used as well as the significant findings of the studies. Additionally, we intended to outline the presence of various self-management components and activities, and to identify gaps in which certain activities were underrepresented or missing.

## Methods

We conducted a scoping review to map the evidence on self-management interventions for haemodialysis patients, identify key study characteristics, and highlight research gaps [23–26]. This approach can serve as a preliminary study for a future systematic review while maintaining methodological rigor [26]. This review was conducted and reported in alignment with the Preferred Reporting Items for Systematic Reviews and Meta-Analyses for Scoping Reviews (PRISMA-ScR) guidelines [27] and followed the Joanna Briggs Institute (JBI) methodology for conducting and reporting scoping reviews [28].

### Literature search

Two researchers (N.K.F. and N.S.) independently conducted a search of six databases (MedLine All, Emcare, CINAHL, PsycINFO, Web of Science, and Cochrane) with support of a librarian for a 6 week period of time in June 2024. Search terms included combinations of thesaurus terms (e.g. Medical Subject Headings (MeSH Terms) in MedLine) and free-text terms using the Participants, Concept and Context (PCC) scheme (23). Free text terms for identifying the target participants (e.g. “ckd patient*” or “end? stage renal failure”) were used in AND-combinations with the concept of self-management (e.g. “self manag*” or “self help“) combined with the search terms relating to the source of evidence (Randomized contolled trials (RCTs)) (e.g. “RCT” or “randomi? ed trial”). As the search was conducted globally and without consideration of cultural background, the ‘context’ component of the PCC framework was not included in the construction of the search string. However, the context, specifically in terms of the healthcare domain and everyday life management, was considered as an inclusion criterion. Available MeSH terms were added, provided they matched the corresponding free text terms. The database searches were restricted to the parameters ‘title’ and ‘abstract’ when these fields were available. For a detailed description of the search strategy, see supplementary file 1.

### Inclusion and exclusion criteria

The inclusion and exclusion criteria are presented in Table 1.

**Table 1:** Inclusion and exclusion criteria based on the PCC* framework

Evidence Sources PCC* Items	Inclusion Criteria	Exclusion Criteria
Evidence Sources	Randomized Controlled Trials (RCTs) published in a peer reviewed journal	Book Chapters, Books, Case-Control Studies, Case Studies, Clinical Trials without randomization, Cohort Studies, Comments, Conference Abstracts, Conference Papers, Feasibility Studies, Grey Literature/Unpublished Literature, Observational Studies, Study protocols or ongoing Studies, Pilot Studies, Posters, Proof of Concept Studies, Quasi-Experimental Studies without a Control Group, Websites (except those used for databases)
Participants	Individuals over 18 years old diagnosed with chronic kidney disease undergoing haemodialysis	Individuals with chronic kidney disease treated with hemofiltration, apheresis, or peritoneal dialysis; Medical personnel
Concept	Self-management theory according to Lorig and Holman (2003), Schäfer-Keller (2009) or related authors; Self-management was part of the program/ intervention title or was listed as the underlying theoretical framework in the background section	Studies focusing exclusively on related concepts or terms (e.g., self-efficacy, self-care) without directly addressing self-management as a core framework, intervention title, or program component No content regarding the provision of information on other renal replacement therapies, such as efforts to enhance knowledge about transplantation
Context	Health care domain/everyday life management; Independent of residence, regardless of cultural background	None

*PCC scheme according to Peters et al. (2021): Participants, Concept, Context

The shift from a medicocentric model to patient-centred care has led to the emergence of the self-management concept, which has been followed by a growing body of literature in this field [29]. Therefore, only articles that incorporated the self-management concept as outlined by Lorig and Holman [15] or related authors were included, either as the underlying theoretical framework or explicitly mentioned in the title or abstract of the intervention or program.

### Data extraction and analysis

Data from the included studies were extracted using a directed content analysis approach based on Hiesieh and Shannon [30] which is commonly used in scoping reviews to simplify complex data [28, 31]. According to the approach used, we organized the data according to pre-identified themes, with additional themes added as they emerged. Our analysis was guided by a self-management model which was developed for patients receiving another renal replacement therapy, namely kidney transplantation [18]. It is based on chronic illness populations [15, 18, 32]. The model focuses on three components (1) ‘managing the medical regimen’, (2) ‘managing emotions and (3) ‘managing (new) life roles’ [18]. Additionally patients have or acquire a set of core skills and self-management activities [15, 18] including 1) ‘problem solving’, 2) ‘decision making’, 3) ‘resource location and skill utilization’ 4) ‘partnership building’ and 5) ‘action planning’. This model already includes specific areas of activities assigned to these components. For example, ‘sun protection’ or ‘symptom management’ were categorized under ‘managing the medical regimen’ (see Table 3). During the analysis, we mapped the content of the included studies against this predefined model. Additionally, where activities specific to the haemodialysis context emerged — such as ‘vascular access management’ — we inductively added these as new self-management components, as displayed in Table 3; Fig. 2.

The content was categorized under these components, with sub-components or new components added as they emerged. Two of the authors (N.K.F. and H.H.) reviewed and reached consensus as they were conducting each step of the process. Study characteristics were summarized in tables.

## Results

**Fig. 1:**
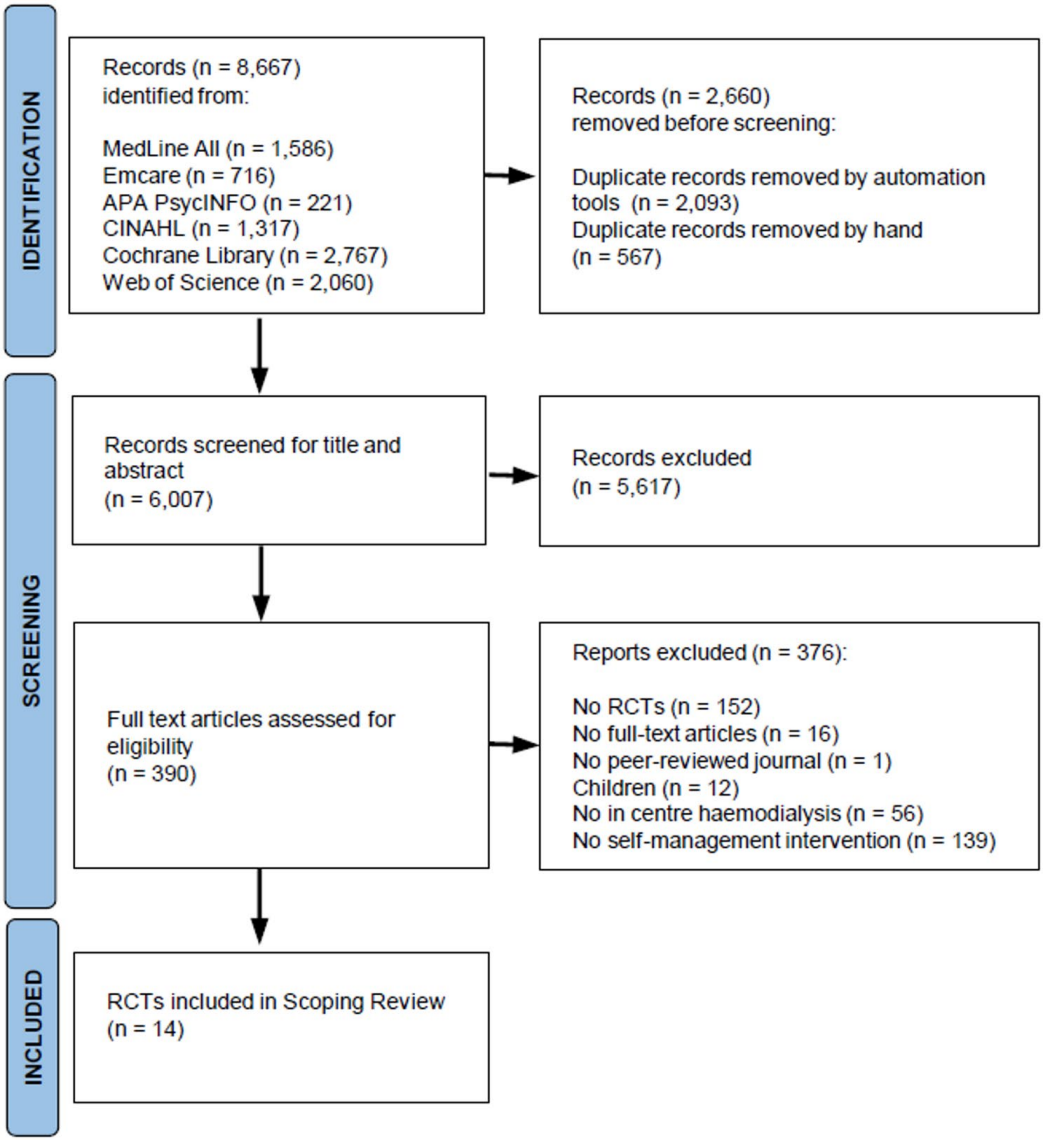
Preferred Reporting Items for Systematic Reviews and Meta-Analyses for Scoping Reviews flow diagram

The search in the databases MedLine All, Emcare, CINAHL, PsycINFO, Web of Science and Cochrane yielded 8,667 records. After removing duplicates, 6,007 articles remained. After title and abstract screening, 390 full texts were assessed for eligibility. Checking the reference lists of eligible articles did not result in any additional articles. Full text review resulted in the exclusion of 376 articles, and remaining 14 articles were eligible for inclusion, describing 13 interventions. Details of the search process are displayed in Fig. 1.

The HED-SMART intervention was assessed in two articles: Griva et al. (2017) [33] investigated the effectiveness of the HED-SMART self-management training program on the effect of adherence, self-management skills, and clinical outcomes. However, Griva and associates (2018) [34] also investigated the long-term development of anxiety and depression in haemodialysis patients.

### Study characteristics

Overall, 14 randomized controlled trials from eight countries were included. An overview is shown in Table 2. The majority of the studies originate from Asia [20, 22, 33–40] (*n* = 10; 71,4%). The shortest study duration was 6 weeks [21, 39], while the longest lasted 21 months [22]. On average, the study duration across all included studies was approximately 5.57 months. Self-management [20, 22, 33, 35, 37, 40] as well as self-efficacy [21, 22, 33, 36, 38, 39] and quality of life [20, 21, 36, 38, 41, 42] were the most frequently investigated study outcomes (each *n* = 6; 37.5%), followed by depressive symptoms [34, 36, 39–41] (*n* = 5; 35.7%). The most used instrument was SUPPH (Strategies Used by People to Promote Health) for measuring self-care self-efficacy (*n* = 3; 21,4%) [21, 36, 39]. The second most commonly used instruments were KDQOL-SF (Kidney Disease Quality of Life Short Form) for measuring quality of life [38, 42], HADS (Hospital Anxiety and Depression Scale) for measuring anxiety and depressive symptoms [34, 40], as well as the BDI (Beck’s Depression Inventory) for measuring depressive symptoms [36, 39] (each *n* = 2; 14,3%).

**Table 2:** Overview of studies, study outcomes their measurements and significant results

Study, year & origin	Sample size, mean age in years & study duration	Study outcomes & Instruments/Measurements	Summary of significant outcomes (without significant subscales or sub dimensions of an instrument)
Keivan et al. 2023 Iran	*n* = 60 Age: 53* Study duration: 3 months	• Quality of life: *Kidney Disease Quality of Life–Short Form (KDQOL–SF)*	Intervention group (IG) vs. control group (CG): • KDQOL-SF scores: ↑ in IG (*p* < 0.05)
Wu et al. 2022 China	*n* = 90 Age: 63* Study duration: 6 months	• Knowledge: *Awareness rate of relevant knowledge of haemodialysis patients developed by the authors* • Compliance: *Treatment Compliance Scale for maintenance haemodialysis patients with end-stage renal disease* • Self-Management: *Self-Management Scale for maintenance haemodialysis patients* • Quality of life: *World Health Organization* (*WHO) Quality of Life-Short Form*	IG vs. CG: • Knowledge awareness rate: ↑ in IG compared to CG (*p* < 0.05) • Treatment Compliance Scores: ↑ in IG compared CG (*p* < 0.05) • Self-Management Scores: ↑ in IG compared to CG (*p* < 0.05) • Quality of Life Scores: ↑ in IG compared to CG (*p* < 0.05)
Zuo et al. 2022 China	*n* = 118 Age: 56 Study duration: 6 months	• Daily steps: *Waistband/belt* • Fatigue: *Revised Piper Fatigue Scale (RPFS)* • Vitality: *Health Survey Questionnaire Vitality Level*,* SF-36 Vitality Level* • Anxiety & depression: *Hospital Anxiety and Depression Scale (HADS)* • Sleep quality: *Pittsburgh Sleep Quality Index (PSQI)* • Social support: *Perceived Social Support Scale (PSSS)* • Self-management of behaviours: *Behavioural Self-Management Scale*	IG vs. CG: • *RPFS*: ↓ in IG compared to CG (*p* = 0.000) • Self-management of behaviours, depression & vitality: improvement in IG compared to CG (*p* < 0.05)
Dingwall et al. 2021 Australia	*n* = 156 Age: 55 Study duration: 6 months	• Psychological distress: *Kessler distress scale; K10* • Depressive symptoms: *Adapted Patient Health Questionnaire; PHQ-9* • Quality of life: *EuroQoL; EQ-5D* • Dialysis Attendance: *Number of missed dialysis sessions*	Within IG (HepB/DSS): • K10 score: ↓ in IG by 3.5 points from baseline to T3 (95% CI: 1.5–5.7; *p* = 0.001) compared to CG • PHQ-9 score: ↓ in IG by 1.6 points from baseline to T3 (95% CI: 0.3–2.9; *p* = 0.03) compared to CG
Li & Yin 2021 Taiwan	*n* = 100 Age: NR Study duration: 3 months	• Self-management: *Patient’s self-management scale developed by the authors* • Internal fistula quality assessment scale: *Developed by the authors* • Thrombus monitoring: *Doppler ultrasound diagnostic*	Intervention vs. control group: • Self-Management scores: ↑ self-management scores (*p* < 0.001) in IG compared to CG • Internal fistula quality: ↑ in IG (T2: 78.5%, T3: 90%) compared to CG (T2: 74%, T3: 79%) (*p* < 0.001)
Pack & Lee 2020 Korea	*n* = 75 Age: 51 Study duration: 3 months	• Biochemical blood markers: Serum Phosphorus, Serum Potassium, Serum Albumin • Self-efficacy: Self-efficacy questionnaire for haemodialysis patients according to Seo et al. 2012 • Quality of Life: Kidney Disease Quality of Life Instrument-Short Form (KDQOL-SF)	• Serum Phosphorus Levels: IG: ↓ by 0.89 points (T0-T3), CG: ↓ by 0.17 points (T0-T3); Significant time × group interaction (F = 50.31, *p* < 0.001); Significant time effect (F = 17.59, *p* < 0.001) and group difference (F = 4.89, *p* = 0.030) • Serum Potassium Levels: IG: ↓ by 0.35 points (T0-T3); CG: ↓ by 0.07 points (T0-T3); Significant time × group interaction (F = 37.01, *p* < 0.001); Significant time effect (F = 101.40, *p* < 0.001) & group difference (F = 5.66, *p* = 0.020) • Self-efficacy: IG: ↑ by 8.14 points (T0-T3); CG: ↑ by 1.58 points (T0-T3); Significant time × group interaction (F = 49.46, *p* < 0.001) • KDQOL-SF: IG: ↑ by 6.67 points (T0-T3); CG: ↓ by 0.21 points (T0-T3); Significant time × group interaction (F = 56.67, *p* < 0.001); Significant time effect (F = 89.73, *p* < 0.001)
Ren et al. 2019 China	*n* = 120 Age: 44 Study duration: 21 months	• Self-management: *The Self-Management Scale for haemodialysis patients according to Song (2009)* • Patient knowledge: *Haemodialysis patient knowledge according to Curtin*,* Sitter*,* Schatell and Chewning (2004)* • Self-efficacy: *Chronic Disease Self-Efficacy Scale (CDSES)*	• Self-management: Within IG: ↑ from T0 to T1 (t = 3.59, *p* < 0.01); Within CG ↑ (t = 2.43, *p* < 0.05) at T2; IG vs. CG: ↑ at T1 (F = 4.13, *p* < 0.05)
Griva et al. 2018 Singapore	*n* = 235 Age: 54 Study duration: 9 months	• Emotional Distress *Hospital Anxiety and Depression Scale (HADS)*	IG vs. CG: • Depression: ↓ depressive symptoms in IG over 12 months (B = − 1.44, SE = 0.64, *p* = 0.03) compared to CG
Griva et al. 2017 Singapore	*n* = 235 Age: 54 Study duration: 9 months	• Biochemical blood markers: *Serum Potassium*,* Phosphate concentrations* • Absolute intradialytic weight gains (IDWG): *Mean pre-to-post dialysis weight change* • Relative intradialytic weight gains: *Absolute IDWG divided by dry weight* • Self-reported adherence: *Renal Adherence Behaviour Questionnaire* • Self-reported adherence to patients’ prescribed medications: *The Medication Adherence Report Scale* • Self-management: *Subscales of the Health Education Impact Questionnaire Version 2* • Self-efficacy: *Self-Efficacy for Managing Chronic Disease Questionnaire*	IG vs. CG: • IDWG: ↓ across all assessments relative to T0 and the usual-care arm at T2 and T3 (*p* < 0.001) HED-SMART Group (IG) only: • Potassium: ↓ from baseline to T3 and T4 (*P* < 0.001). • Phosphate: Improvement from baseline to T3 (*p* = 0.03)
Liu et al. 2016 China	*n* = 86 Age: 43* Study duration: 6 months	• Disease related knowledge: *“Patient’s grasp of disease knowledge” according to Cheng (2005)* • Self-management: *“Parameters of chronic disease self* *management behavior” according to Lorig*,* Stewart and Ritter (2009) & Lorig (2003)*	IG vs. CG: • Disease-related knowledge: Improvement compared to baseline and control group (*p* < 0.05). Within intervention group: • Disease related knowledge: ↑ scores compared with baseline (*p* < 0.05)
Karavetian & Ghaddar 2012 Lebanon	*n* = 122 Age: 58 Study duration: 8 weeks	• Biochemical blood markers: *Biochemical parameters (Serum Phosphorus*,* Calcium and Phosphate product (CA x P)* • Patient knowledge (PK): *Patient knowledge questionnaire*,* adapted from Ford et al. 2004* • Dietary non-adherence: *Patient dietary non-adherence (PDnA) to phosphate restricted diet questionnaire*,* adapted format of the School Physical Activity and Nutrition (SPAN) questionnaire (Hoelscher et al. 2003)*	Within IG (group A) over time: • Serum phosphorus levels: Improvement (*p* = 0.01) • Patient knowledge scores: Improvement (*p* = 0.02) • Dietary non-adherence: Improvement (*p* = 0.01) IGs (group A & group B) vs. CG (group C): • Serum Ca x P product: Improvement in IGs A (*p* = 0.006) and intervention group B (*p* = 0.01)
Moattari et al. 2012 Iran	*n* = 48 Age: 38 Study duration: 6 weeks	• Self-care & self-efficacy: *Strategies Used by People to Promote Health (SUPPH)* • Quality of Life: *QoL questionnaire according to Estwing Ferrans and Marjorie Powers (1985)* • Blood pressure: *Standardized blood pressure pre dialysis of two measurements on two consecutive Haemodialysis sessions* • Intradialytic weight gain (IDWG): *Amount of weight gain between the end of one dialysis session and the beginning of the next* • Biochemical blood markers: *Sodium*,* potassium*,* creatinine*,* blood urea nitrogen*,* phosphorous*,* calcium*,* hemoglobin and hematocrit*	IG vs. CG: • SUPPH: ↑ by 12.02 points in IG compared to CG (*p* < 0.001) • QoL score: ↑ by 2.93 points in IG compared to CG (*p* < 0.001) • IDWG: ↓ by 0.44 points in IG compared to CG (*p* < 0.0039) • Hemoglobin: ↓ by 1.99 points in the IG compared to CG (*p* < 0.005) • Heamatocrit: ↓ by 6.4 in the IG compared to CG (*p* < 0.004) • Systolic blood pressure: ↓ by 14.35 points in the IG compared to CG (*p* < 0.001) • Diastolic blood pressure: ↓ by 5.62 points in the experimental group compared to control group (*p* < 0.003)
Lii et al. 2006 Taiwan	*n* = 60 Age: NR Study duration: 2 months	• Self-care self-efficacy: *Strategies Used by People to Promote Health (SUPPH) * • Depression: *Beck’s Depression Inventory (BDI)* • Quality of life: *The Medical Outcomes Study 36-Item Short Form (SF-36)*	IG vs. CG: • SUPPH: ↑ in IG (16.00, SD = 11.32) compared to CG (-6.89, SD = 14.24) (t = 5.96, *p* < 0.001) • BDI: ↓ in IG (-3.05, SD = 9.16) compared to ↑ in CG (9.21, SD = 13.17) (t = -3.81, *p* < 0.001)
Tsay & Hung 2004 Taiwan	*n* = 50 Age: 51 Study duration: 6 weeks	• Empowerment: *Modified Empowerment Scale (ES)* • Self-care self-efficacy: *Strategies Used by People to Promote Health (SUPPH)* • Depression: *Beck Depression Inventory (BDI)*	IG vs. CG: • ES: ↑ in IG compared to CG (t(48) = 6.54, *p* < 0.001) • SUPPH: ↑ self-care self-efficacy scores in IG compared to CG after adjusting (F(1,47) = 10.82, MSE = 10.08, *p* = 0.002, partial η² = 0.19) • BDI: ↓ in IG compared to CG (t(48) = 2.49, *p* = 0.02)

Abbreviations: BDI: Beck Depression Inventory, CA x P: Calcium and Phosphate product, CDSES: Chronic Disease Self-Efficacy Scale, CG: Control group, ES: Modified Empowerment Scale, HADS: Hospital Anxiety and Depression Scale, HED-SMART: Hemodialysis Self-management Randomized Trial, IDWG: Intradialytic weight gain, IG: Intervention group, K10: Kessler distress scale, KDQOL-SF: Kidney Disease Quality of Life Instrument-Short Form, MSE: Modified Empowerment Scale, PDnA: Patient dietary non-adherence, PHQ-9: Patient Health Questionnaire, PK: Patient knowledge, PSSS: Perceived Social Support Scale, PSQI: Pittsburgh Sleep Quality Index, QoL: Quality of Life, RPFS: Revised Piper Fatigue Scale, SD: Standard Deviation, SF-36: The Medical Outcomes Study 36-Item Short Form, SUPPH: Self-care self-efficacy, SPAN: School Physical Activity and Nutrition, NR: Not Reported, WHO: World Health Organization

*data were calculated from the available data of the study

Statistically significant results were reported in all of the 14 studies, particularly in improvements related to depressive symptoms (*n* = 5; 35.7%) [34, 36, 39–41], biochemical blood markers like serum potassium and phosphate [21, 33, 38, 43], and self-efficacy [21, 36, 38, 39] (each *n* = 4; 28,6%). Additional study characteristics can be seen in supplementary Table.

### Content analysis of studies on self-management components and activities

**Fig. 2:**
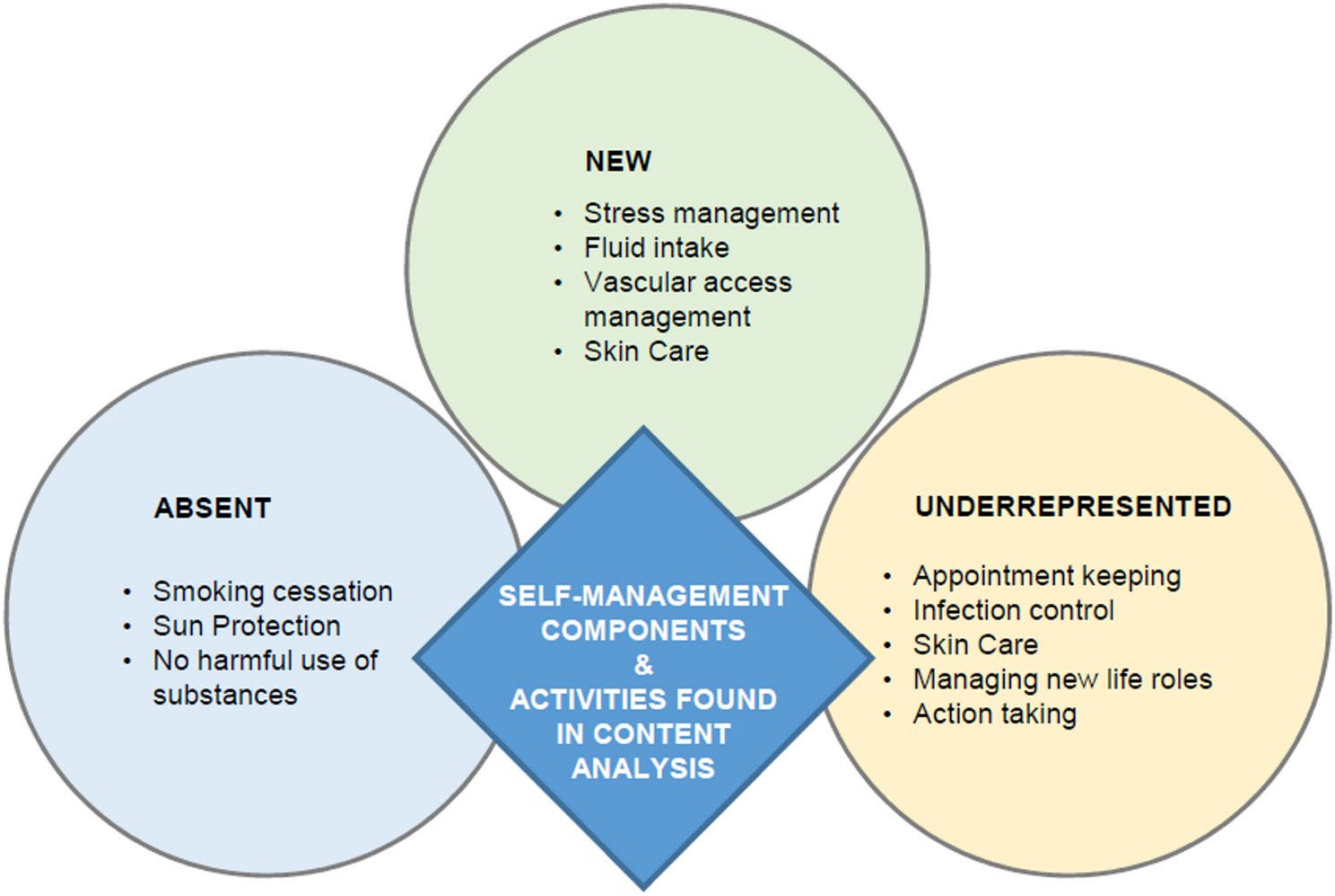
Self-management components and activities for haemodialysis patients: New, absent, and underrepresented elements

The studied interventions included different components and activities of self-management in managing everyday life for haemodialysis patients. An overview can be seen in Table 3. The most frequently studied self-management components and activities were ‘diet’ [20–22, 33, 34, 37, 38, 40, 42, 43] (n = 10; 71.4%), ‘managing emotions’ [20–22, 36, 37, 39, 41] (n = 7; 50%), and ‘medication taking’ [21, 33, 34, 37, 42, 43] (n = 6; 42.9%). However, other components and activities, such as ‘infection prevention’ [35], ‘appointment keeping’ [21] and ‘managing new life roles’ [21] (each n = 1; 7.1%) were notably underrepresented. In this context, ‘underrepresented’ was defined as components and activities that were identified in only one of the 14 included studies. Additionally, ‘smoking cessation’, ‘sun protection’ and ‘decision making’ are absent from the studies reviewed. At the same time, we identified new components and activities within the studies that were not present in the model used, but are particularly significant for haemodialysis patients. These include ‘vascular access management’ [20, 35, 37, 42], ‘managing complications’ [35, 37, 40, 42], e.g. in the event of bleeding from the vascular access, ‘skin care’ [42], which is relevant to pruritus (itching), and ‘stress-management’ [36, 39] to cope with the psychological stress due to the long duration of treatment, physical limitations and the unpredictability of their health situation [36, 39]. Several studies examined multiple components and activities of self-management simultaneously, while others focused on just one or a few specific aspects. 78.6% (*n* = 11) of studies included four or more components and activities in their intervention [20–22, 33–37, 39, 40, 42]. For example, studies like Karavetian & Ghaddar [43] and Wu et al. [20] covered a broad range of components and activities. In contrast, other studies, such as Dingwall et al. [41] focused on fewer components and activities. Figure 2 summarizes the new, as well as the underrepresented and absent components and activities of self-management.

**Table 3:** Overview of studies according to self-management components in haemodialysis patients

**Self-management components**	**Studies**														
	Keivan et al. 2023	Wu et al. 2022	Zuo et al. 2022	Dingwall et al. 2021	Li & Yin 2021	Pack & Lee 2020	Ren et al. 2019	Griva et al. 2018	Griva et al. 2017	Liu et al. 2016	Karavetian & Ghaddar 2012	Moattari et al. 2012	Lii et al. 2006	Tsay & Hung 2003	**Count of studies**
**Managing the medical regimen**															
Diet*	✔	✔	✔	-	-	✔	✔	✔	✔	✔	✔	✔	-	-	10
Medication taking	✔	-	-	-	-	-	-	✔	✔	✔	✔	✔	-	-	6
Fluid intake^1^	✔	-	✔	-	-	-	✔	✔	✔	-	-	-	-	-	5
Symptom management	-	✔	✔	-	-	-	-	-	-	✔	-	-	✔	-	4
Managing complications^1^	✔	-	✔	-	✔	-	-	-	-	✔	-	-	-	-	4
Vascular access management^1^	✔	✔	-	-	✔	-	-	-	-	✔	-	-	-	-	4
Monitoring vital signs	✔	-	-	-	✔	-	-	-	-	✔	-	-	-	-	3
Physical exercising	✔	-	✔	-	-	-	-	-	-	✔	-	-	-	-	3
Stress management^1^	-	-	-	-	-	-	-	-	-	-	-	-	✔	✔	2
Coping with staff^1^	-	✔	-	-	-	-	-	-	-	-	-	✔	-	-	2
Appointment keeping	-	-	-	-	-	-	-	-	-	-	-	✔	-	-	1
Infection control	-	-	-	-	✔	-	-	-	-	-	-	-	-	-	1
Skin care^1^	✔	-	-	-	-	-	-	-	-	-	-	-	-	-	1
Smoking cessation	-	-	-	-	-	-	-	-	-	-	-	-	-	-	0
Sun protection	-	-	-	-	-	-	-	-	-	-	-	-	-	-	0
No harmful use of substances	-	-	-	-	-	-	-	-	-	-	-	-	-	-	0
**Managing emotions**															
Managing emotions	-	✔	-	✔	-	-	✔	-	-	✔	-	✔	✔	✔	7
**Managing new life roles**															
Managing new life roles	-	-	-	-	-	-	-	-	-	-	-	✔	-	-	1
**Core skills**															
Resource finding and utilization	-	-	-	✔	-	-	✔	✔	✔	-	-	-	-	-	4
Partnership making	-	✔	-	✔	-	-	-	-	-	-	-	✔	-	✔	4
Problem solving	-	-	-	-	-	-	-	-	-	-	-	✔	✔	✔	3
Decision making	-	-	-	-	-	-	-	-	-	-	✔	-		✔	2
Action taking	-	✔	-	-	-	-	-	-	-	-	-	-	-	-	1
**Count of targeted self-management components**	8	7	5	3	4	1	4	4	4	8	3	8	4	5	

**Legend**: ^*^The term “healthy eating” was adapted to “diet” in the context of haemodialysis patients; ^1^ New dimensions identified through the literature analysis that are relevant for haemodialysis patients

## Discussion

This scoping review provides a comprehensive overview of self-management interventions for haemodialysis patients, highlighting their broad thematic scope, common intervention outcomes, and key self-management components. By synthesizing findings from fourteen studies across eight countries, we identified frequently measured outcomes such as quality of life, self-management, and self-efficacy, along with the most commonly used instruments. The majority of studies originated from Asia, which can be explained by several factors. Asian countries are known for their healthcare systems offering extensive coverage for dialysis treatments [44, 45]. Furthermore, these nations often prioritize preventive care, which promotes a focus on self-management strategies [46]. Additionally, many Asian governments invest in research on chronic diseases, including chronic kidney disease and dialysis treatment, due to the substantial financial burden these conditions impose on the healthcare system [45, 47].

The studies have shown that haemodialysis patients face numerous challenges in managing their treatment, including adhering to a strict regimen involving diet, fluid restrictions, and a demanding dialysis schedule [4, 5, 48]. These difficulties often lead to inadequate self-management, which can affect key outcomes like self-efficacy and quality of life - both crucial factors for improving long-term results [49]. Our scoping review has shown that these outcomes, along with self-management, are the most frequently investigated (37.5%). This reflects that the evidence presented here highlights the essential role of those outcomes in addressing complications and mental health burdens faced by haemodialysis patients.

Adding to another a scoping review by Donald et al. [50] we found that most of the studies defined multiple outcomes rather than just a single primary outcome. However, in their 2018 scoping review, Donald et al. [50] observed that most self-management interventions for adults with chronic kidney disease primarily focused on physiological outcomes, such as blood pressure. In contrast, we found that symptoms measured through Patient-Reported Outcome Measures (PROMs), with SUPPH being the most frequently used tool for assessing self-care self-efficacy, were more frequently addressed in the interventions we reviewed. The frequent use of standardized tools as the KDQOL-SF, HADS, and BDI underscores the multidimensional nature of self-management, covering both quality of life and psychological well-being. Notably, our review included seven studies published after 2018, which may indicate a shift in research focus. This difference can be explained by a general trend highlighted by the growing emphasis on the use of PROMs, as healthcare authorities and researchers increasingly recognize their vital role in assessing care quality from the patient’s perspective [51]. However, our findings indicate a lack of a universally accepted patient reported outcome measurement for assessing self-management in haemodialysis patients. While self-management was measured in six [20, 22, 33, 35, 37, 40] of the fourteen included studies, different measurement tools were used, highlighting the absence of a standardized approach for this specific population. This variability complicates international comparisons and underscores the need for a widely accepted instrument to ensure consistency and comparability across studies.

This shift in focus towards PROMs aligns with the broader trend of recognizing self-management as a central concept in chronic illness care. This perspective underscores the importance of empowering patients and tailoring care to their specific needs. Notably, the majority of the studies implemented four or more components in their self-management programs, highlighting a clear emphasis on comprehensive approaches using intervention bundles instead of singular interventions. This approach acknowledges the complex and multifaceted needs of haemodialysis patients, demonstrating that effective self-management requires support across several dimensions of care [14, 15, 52, 53]. Beyond enhancing effectiveness, incorporating multiple components and activities within a self-management plan aligns with patients’ interests and preferences, fostering their active participation and empowerment [54].

While the need for incorporating several self-management components in an intervention seems to be practiced widely, the specific content of self-management interventions had a broad range. When examining the content of the interventions, the majority of studies focused on dimensions like ‘diet’ and ‘managing emotions’, while other elements such as ‘infection prevention’, ‘appointment adherence’, and ‘managing new life roles’ were underrepresented. Notably, dimensions such as ‘smoking cessation’, ‘sun protection’ and ‘decision-making’ were absent from the reviewed literature and clearly point for a need for future studies to fill this gap.

Although underrepresented or absent in the researched studies, it does not mean that these self-management components and activities are irrelevant for haemodialysis patients. For example, infection prevention is crucial, as infections are the second leading cause of death in this population [55]. The COVID-19 pandemic marked an increase in overall mortality among the haemodialysis patients [55] highlighting the urgent need to incorporate additional infection prevention strategies into haemodialysis patient self-management. A similar case applies to the missing self-management dimensions. For instance, ‘smoking cessation’ was not identified as a dimension in any of the studies reviewed. However, the literature shows that dialysis patients who smoke ‘are more hypertensive, more fluid overloaded, and take more antihypertensive medications than non-smokers’ [56]. This underscores the urgent need to incorporate these dimensions into future studies that address the underrepresented or absent aspects of self-management in haemodialysis patients. Future research should incorporate these overlooked components and activities to develop a more comprehensive approach to supporting self-management in this population. Doing so could empower patients in practice to take greater control over their health decisions [57].

### Strengths and limitations

This scoping review reveals several strengths and limitations. First, a strength of this study is that it was grounded on a theoretical model as were the definition and operationalization of the concept of self-management interventions for haemodialysis patients. To our knowledge, this is the first study that combined this approach with a comprehensive content analysis following scoping review guidelines.

Second, a comprehensive overview of the outcomes of self-management interventions, their corresponding measurement tools as well as their significant results were conducted. This allowed identification of the quantity and diversity of tools to measure self-management outcomes, such as quality of life, self-management, and self-efficacy. The findings from this review form a foundation for further research aimed at standardizing outcome measures to ensure that interventions can be more reliably compared across different settings and populations.

At the same time, the comprehensive content analysis assessed new, underrepresented or absent components and activities of self-management interventions for haemodialysis patients. This can serve as a valuable foundation for guiding the development of future self-management interventions and programs.

However, this study included haemodialysis patients treated across various geographic locations worldwide, which brings with it variations in dialysis treatment settings that can influence patient care and outcomes. For instance, in the United States, haemodialysis is predominantly performed in ambulatory dialysis centres, whereas in countries like China the treatment is mostly provided in hospitals or hospital-affiliated dialysis centres. This contextual difference in the treatment setting was not specifically addressed in the analysis, despite its potential impact on patient outcomes.

Additionally, the inclusion criteria of studies were limited. On the one hand, it was restricted to studies that explicitly studied self-management as a theory or intervention. However, the relationship between self-management and related concepts, such as self-care was not always clear. Since self-management is often conceptualized as a subset of self-care [52, 58, 59], the focus of our study may not fully capture the range of interventions available. This could mean that the broader spectrum of self-care interventions for haemodialysis that potentially also influenced self-management were missed, limiting the comprehensiveness of our findings. On the other hand, this review focuses exclusively on self-management interventions in adults, which may limit the generalizability of findings to younger populations, as needs and challenges may differ significantly.

Moreover, it was sometimes challenging to assess the exact content of interventions in some of the studies due to insufficient or inconsistent reporting. This lack of detailed description made it difficult to thoroughly understand the components of the interventions and evaluate their relevance to self-management. As a result, important nuances regarding the interventions’ design and implementation may have been missed.

Furthermore, it is important to acknowledge that the approach of a scoping review is primarily descriptive in nature and does not allow for conclusions regarding effect sizes or causal relationships. The focus is instead on mapping and synthesizing the available evidence to provide a comprehensive overview of the research field. While our exclusive focus on randomized controlled trials (RCTs) ensures a high level of methodological rigor and supports evidence-based intervention development, it may have excluded valuable insights from other study designs. Qualitative studies or observational research, for example, might provide a deeper understanding of patient experiences and contextual factors relevant to self-management.

## Conclusion

This innovative scoping review provides the first comprehensive overview of existing evidence from randomized controlled trials (RCTs) on self-management interventions for haemodialysis patients. Overall, fourteen articles from eight countries were included. We presented the investigated outcomes, their corresponding measurement instruments, and significant findings for each study. Most frequent intervention outcomes were ‘quality of life’, ‘self-management' and ‘self-efficacy’. The most used instrument was SUPPH (Strategies used by people to promote health) for measuring self-care self-efficacy. All authors of the included studies reported significant results. Additionally, we conducted a content analysis to assess the inclusion of different self-management components and activities within the interventions. It identified commonly addressed self-management components and activities for haemodialysis patients, such as ‘emotion regulation,’ ‘medication adherence’ and ‘diet management’. We highlighted three categories of self-management components and activities that were found in the literature. New (e.g. ‘stress-management‘), as well as underrepresented (e.g. ‘infection-control’) and absent (e.g. ‘smoking-cessation’) self-management components and activities were identified. This scoping review highlights the need for a comprehensive and conceptually grounded approach to self-management interventions, including underrepresented or absent elements to better support haemodialysis patients. Additionally future studies should support standardized outcome measurement, to ensure self-management interventions are both targeted and comparable across contexts.

## Electronic supplementary material

Below is the link to the electronic supplementary material.

Supplementary Material 1

Supplementary Material 2

## Data Availability

No datasets were generated or analysed during the current study.

## Abbreviations

BDI: Beck Depression Inventory
CA x P: Calcium and Phosphate product
CDSES: Chronic Disease Self-Efficacy Scale
CG: Control Group
CKD: Chronic Kidney Disease
ES: Modified Empowerment Scale
HADS: Hospital Anxiety and Depression Scale
IDWG: Intradialytic weight gain
IG: Intervention Group
JBI: Joanna Briggs Institute
K10: Kessler distress scale
KDQOL-SF: Kidney Disease Quality of Life Instrument-Short Form
MeSH: Medical Subject Headings
PCC: Participants, Concept, Context
PDnA: Patient dietary non-adherence
PHQ-9: Patient Health Questionnaire
PK: Patient knowledge
PRISMA-ScR: Preferred Reporting Items for Systematic Reviews and Meta-Analyses for Scoping Reviews
PROM: Patient Reported Outcome Measures
PSSS: Perceived Social Support Scale
PSQI: Pittsburgh Sleep Quality Index
QoL: Quality of Life
RCT: Randomised Controlled trials
RPFS: Revised Piper Fatigue Scale
SF-36: The Medical Outcomes Study 36-Item Short Form
SUPPH: Self-care self-efficacy
SPAN: School Physical Activity and Nutrition
WHO: World Health Organization
