# Supplementary material for: Self-management interventions for adult haemodialysis patients: a scoping review of randomized controlled trials
Source: BMC Nephrol. 2025 Jun 13;26:285. doi: 10.1186/s12882-025-04229-6 (PMC12166570; doi:10.1186/s12882-025-04229-6)
Supplement: Supplementary file 1 — Supplementary Material 1 [file 12882_2025_4229_MOESM1_ESM.docx]

**Supplementary**

Table S.1. Search strings for MedLine All (OVID), Emcare (OVID), APA PsycINFO (EBSCO), CINAHL (EBSCO), Cochrane Library (Wiley), Web of Science Core Collection (Clarivate)

**Table S.1.** Search strings for CENTRAL, Embase, MEDLINE/PubMed and PsycINFO

|  | MedLine All | Emcare | CINAHL | PsycINFO | Web of Science | Cochrane |
| --- | --- | --- | --- | --- | --- | --- |
| 1 | dialys?s.ti,ab,kf. | dialys?s.ti,ab,kf. | AB dialys?s OR TI dialys?s | AB dialys?s OR TI dialys?s | dialys?s | dialys?s |
| 2 | hemodialys?s.ti,ab,kf. | hemodialys?s.ti,ab,kf. | AB hemodialys?s OR TI hemodialys?s | AB hemodialys?s OR TI hemodialys?s | hemodialys?s | hemodialys?s |
| 3 | ckd.ti,ab,kf. | ckd.ti,ab,kf. | AB ckd OR TI ckd | AB ckd OR TI ckd | ckd | ckd |
| 4 | ckd patient*.ti,ab,kf. | ckd patient*.ti,ab,kf. | AB ckd patient* OR TI ckd patient* | AB ckd patient* OR TI ckd patient* | ckd patient* | ckd patient* |
| 5 | chronic kidney disease.ti,ab,kf. | chronic kidney disease.ti,ab,kf. | AB chronic kidney disease OR TI chronic kidney disease | AB chronic kidney disease OR TI chronic kidney disease | chronic kidney disease | chronic kidney disease |
| 6 | chronic kidney failure.ti,ab,kf. | chronic kidney failure.ti,ab,kf. | AB chronic kidney failure OR TI chronic kidney failure | AB chronic kidney failure OR TI chronic kidney failure | chronic kidney failure | chronic kidney failure |
| 7 | chronic renal failure.ti,ab,kf. | chronic renal failure.ti,ab,kf. | AB chronic renal failure OR TI chronic renal failure | AB chronic renal failure OR TI chronic renal failure | chronic renal failure | chronic renal failure |
| 8 | end?stage renal disease.ti,ab,kf. | end?stage renal disease.ti,ab,kf. | AB end?stage renal disease OR TI end?stage renal disease | AB end?stage renal disease OR TI end?stage renal disease | end?stage renal disease | end?stage renal disease |
| 9 | end?stage renal failure.ti,ab,kf. | end?stage renal failure.ti,ab,kf. | AB end?stage renal failure OR TI end?stage renal failure | AB end?stage renal failure OR TI end?stage renal failure | end?stage renal failure | end?stage renal failure |
| 10 | end?stage kidney disease.ti,ab,kf. | end?stage kidney disease.ti,ab,kf. | AB end?stage kidney disease OR TI end?stage kidney disease | AB end?stage kidney disease OR TI end?stage kidney disease | end?stage kidney disease | end?stage kidney disease |
| 11 | esrd.ti,ab,kf. | esrd.ti,ab,kf. | AB esrd OR TI esrd | AB esrd OR TI esrd | esrd | esrd |
| 12 | exp Kidney Failure, Chronic/ | exp Kidney Failure, Chronic/ | MH "Kidney Failure, Chronic+" | - | - | MH Kidney Failure, Chronic |
| 13 | exp Renal Dialysis/ | exp Renal Dialysis/ | MH "Renal Dialysis+" | - | - | MH Renal Dialysis |
| 14 | 1 or 2 or 3 or 4 or 5 or 6 or 7 or 8 or 9 or 10 or 11 or 12 or 13 | 1 or 2 or 3 or 4 or 5 or 6 or 7 or 8 or 9 or 10 or 11 or 12 or 13 | 1 or 2 or 3 or 4 or 5 or 6 or 7 or 8 or 9 or 10 or 11 or 12 or 13 | 1 or 2 or 3 or 4 or 5 or 6 or 7 or 8 or 9 or 10 or 11 | TI=(1 or 2 or 3 or 4 or 5 or 6 or 7 or 8 or 9 or 10 or 11) or AB=(1 or 2 or 3 or 4 or 5 or 6 or 7 or 8 or 9 or 10 or 11) | 1 or 2 or 3 or 4 or 5 or 6 or 7 or 8 or 9 or 10 or 11 or 12 or 13 |
| 15 | self car*.ti,ab,kf. | self car*.ti,ab,kf. | AB self car* OR TI self car* | AB self car* OR TI self car* | self car* | self car* |
| 16 | self manag*.ti,ab,kf. | self manag*.ti,ab,kf. | AB self manag* OR TI self manag* | AB self manag* OR TI self manag* | self manag* | self manag* |
| 17 | patient education.ti,ab,kf. | patient education.ti,ab,kf. | AB patient education OR TI patient education | AB patient education OR TI patient education | patient education | patient education |
| 18 | health literacy.ti,ab,kf. | health literacy.ti,ab,kf. | AB health literacy OR TI health literacy | AB health literacy OR TI health literacy | health literacy | health literacy |
| 19 | counselling.ti,ab,kf. | counselling.ti,ab,kf. | AB counselling OR TI counselling | AB counselling OR TI counselling | counselling | counselling |
| 20 | self help.ti,ab,kf. | self help.ti,ab,kf. | AB self help OR TI self help | AB self help OR TI self help | self help | self help |
| 21 | self monitor*.ti,ab,kf. | self monitor*.ti,ab,kf. | AB self monitor* OR TI self monitor* | AB self monitor* OR TI self monitor* | self monitor* | self monitor* |
| 22 | self administration.ti,ab,kf. | self administration.ti,ab,kf. | AB self administration OR TI self administration | AB self administration OR TI self administration | self administration | self administration |
| 23 | behavio?r.ti,ab,kf. | behavio?r.ti,ab,kf. | AB behavio?r OR TI behavio?r | AB behavio?r OR TI behavio?r | behavio?r | behavio?r |
| 24 | behavio?r change.ti,ab,kf. | behavio?r change.ti,ab,kf. | AB behavio?r change OR TI behavio?r change | AB behavio?r change OR TI behavio?r change | behavio?r change | behavio?r change |
| 25 | Self Care/ | Self Care/ | MH "self care+" | MH "self care+" | - | MH Self Care |
| 26 | Self-Management/ | Self-Management/ | MH "Self-Management+" | MH "Self-Management+" | - | MH Self-Management |
| 27 | Patient Education as Topic/ | Patient Education as Topic/ | MH "patient education as topic+" | MH "patient education as topic+" | - | MH Patient Education as Topic |
| 28 | exp Health literacy/ | exp Health literacy/ | MH "Health literacy+" | MH "Health literacy+" | - | MH Health literacy |
| 29 | Self Administration/ | Self Administration/ | MH "self administration+" | MH "self administration+" | - | MH Self Administration |
| 30 | Health Behavior/ | Health Behavior/ | MH "health behavior+" | MH "health behavior+" | - | MH Health Behavior |
| 31 | 15 or 16 or 17 or 18 or 19 or 20 or 21 or 22 or 23 or 24 or 25 or 26 or 27 or 28 or 29 or 30 | 15 or 16 or 17 or 18 or 19 or 20 or 21 or 22 or 23 or 24 or 25 or 26 or 27 or 28 or 29 or 30 | 15 or 16 or 17 or 18 or 19 or 20 or 21 or 22 or 23 or 24 or 25 or 26 or 27 or 28 or 29 or 30 | 15 or 16 or 17 or 18 or 19 or 20 or 21 or 22 or 23 or 24 or 25 or 26 or 27 or 28 or 29 or 30 | TI=(15 or 16 or 17 or 18 or 19 or 20 or 21 or 22 or 23 or 24) or AB=(15 or 16 or 17 or 18 or 19 or 20 or 21 or 22 or 23 or 24) | 15 or 16 or 17 or 18 or 19 or 20 or 21 or 22 or 23 or 24 or 25 or 26 or 27 or 28 or 29 or 30 |
| 32 | randomi?ed trial.ti,ab,kf. | randomi?ed trial.ti,ab,kf. | AB randomi?ed trial OR TI randomi?ed trial | AB randomi?ed trial OR TI randomi?ed trial | randomi?ed trial | randomi?ed trial |

| 33 | controlled clinical trial*.ti,ab,kf. | controlled clinical trial*.ti,ab,kf. | AB controlled clinical trial* OR TI controlled clinical trial* | AB controlled clinical trial* OR TI controlled clinical trial* | controlled clinical trial* | controlled clinical trial* |
| --- | --- | --- | --- | --- | --- | --- |
| 34 | RCT.ti,ab,kf. | RCT.ti,ab,kf. | AB RCT OR TI RCT | AB RCT OR TI RCT | RCT | RCT |
| 35 | randomi?ed controlled trial.ti,ab,kf. | randomi?ed controlled trial.ti,ab,kf. | AB randomi?ed controlled trial OR TI randomi?ed controlled trial | AB randomi?ed controlled trial OR TI randomi?ed controlled trial | randomi?ed controlled trial | randomi?ed controlled trial |
| 36 | random*.ti,ab,kf. | random*.ti,ab,kf. | AB random* OR TI random* | AB random* OR TI random* | random* | random* |
| 37 | trial.ti,ab,kf. | trial.ti,ab,kf. | AB trial OR TI trial | AB trial OR TI trial | trial | trial |
| 38 | intervention*.ti,ab,kf. | intervention*.ti,ab,kf. | AB intervention OR TI Intervention* | AB intervention* OR TI Intervention* | intervention* | intervention* |
| 39 | Randomized Controlled Trials as Topic/ | Randomized Controlled Trials as Topic/ | MH Randomized Controlled Trials as Topic | MH Randomized Controlled Trials as Topic | - | MH Randomized Controlled Trials as Topic |
| 40 | Clinical Trials as Topic/ | Clinical Trials as Topic/ | MH Clinical Trials as Topic | MH Clinical Trials as Topic | - | MH Clinical Trials as Topic |
| 41 | Randomized Controlled Trial.pt. | - | - | - | - | - |
| 42 | Clinical Trial.pt. | - | - | - | - | - |
| 43 | 32 or 33 or 34 or 35 or 36 or 37 or 38 or 39 or 40 or 41 or 42 | 32 or 33 or 34 or 35 or 36 or 37 or 38 or 39 or 40 | 32 or 33 or 34 or 35 or 36 or 37 or 38 or 39 or 40 | 32 or 33 or 34 or 35 or 36 or 37 or 38 or 39 or 40 | TI=(32 or 33 or 34 or 35 or 36 or 37 or 38 or 39 or 40) or AB=(32 or 33 or 34 or 35 or 36 or 37 or 38 or 39 or 40) | 32 or 33 or 34 or 35 or 36 or 37 or 38 or 39 or 40 |
| 44 | 14 and 31 and 43 | 14 and 31 and 43 | 14 and 31 and 43 | 14 and 31 and 43 | 14 and 31 and 43 | 14 and 31 and 43 |

*Note*. * = searches for word variations with different suffixes. $<n> = restricts number of characters following the word. AB/ab = abstract. adj = positional operator which finds two terms next to each other in the specified order. adj3 = positional operator which finds terms in any order with two words (or fewer) between them. exp = explode (retrieve results using the selected thesaurus term and all of its more specific terms). id = key concepts. kw = keyword. md = methodology. pt = publication type. TI/ti = title.
