# Supplementary material for: Self-management interventions for adult haemodialysis patients: a scoping review of randomized controlled trials
Source: BMC Nephrol. 2025 Jun 13;26:285. doi: 10.1186/s12882-025-04229-6 (PMC12166570; doi:10.1186/s12882-025-04229-6)
Supplement: Supplementary file 2 — Supplementary Material 2 [file 12882_2025_4229_MOESM2_ESM.docx]

**Supplementary**

Table S.2. Overview of studies, sample size, group number and study aim

| **Table S.2.:** Overview of studies, sample size, group number and study aim | | | |  |  |  |
| --- | --- | --- | --- | --- | --- | --- |
| **Study, year & origin** | **Sample size, mean age in years, time since initiation of hemodialysis (TSI-HD)** | **Group number** | **Study Aim** | **Delivery format** | **Providers** | **Data collection points** |
| Keivan et al. 2023 Iran | n = 60 Age: 52,7*  TSI-HD: NR | 2 (1 intervention group, 1 control group) | To investigate the role of a self-management program based on the 5 A nursing model in the quality of life of patients undergoing hemodialysis comparing across an intervention and control group | • Individual face-to-face  • Phone calls  • Text messages | • Researcher | T1 = baseline, T2 = 3 months post-intervention |
| Wu et al. 2022 China | n = 90 Age: 63.1*  TSI-HD:21.8 (months) | 2 (1 intervention group, 1 control group) | To investigate the effect of self-determination theory on awareness of relevant knowledge, treatment compliance, self-management level in maintenance hemodialysis comparing an intervention and control group | • Individual face-to-face • Presentations • Written materials | • Nurses | T1 = baseline T2 = 6 months post-intervention |
| Zuo et al. 2022 China | n = 118 Age: 56.39  TSI-HD: NR | 2 (1 intervention group, 1 control group) | To investigate the interaction between nurses and multidisciplinary of nonpharmacological integrated care interventions (NICIs) and assess the impact of fatigue on patients undergoing hemodialysis comparing an intervention and control group | • Individual face-to-face • Presentations • Written materials | • Nurses • Multidisciplinary team | T1 = baseline T2 = 6 months post-intervention |
| Dingwall et al. 2021 Australia | n = 156 Age: 55  TSI-HD: 3.1 years (median) | 3 (2 intervention groups, 1 control group | To investigate the efficacy of psychosocial interventions for Indigenous Australian people with End-Stage Kidney Disease (ESKD) using the "Stay Strong App" (ISS group) comparing to "Hep B Story App" (HepB/DSS group) and usual care (TAU/DSS group) | • Application based • Online interview face-to face • Text messages/phone calls | • Trained researchers | T1 = baseline T2 = 3 months T3 = 6 months |
| **Table S.2. (continued)** |  |  |  |  |  |  |
| **Study, year & origin** | **Sample size, mean age in years, time since initiation of hemodialysis (TSI-HD)** | **Group number** | **Study Aim** | **Delivery format** | **Providers** | **Data collection points** |
| Li et al. 2020 Taiwan | n = 100  Age: NR  TSI-HD: NR | 2 (1 intervention group, 1 control group) | To investigate the impact of a self-management approach on hemodialysis in patients with autogenous arteriovenous fistulas, and evaluate the enhancement of their self-care, along with their ability to manage and protect themselves through this approach, ultimately aiming to enhance the quality of dialysis. | • Individual face-to-face | • Nurses | T1 = baseline, T2 = 1 month post-intervention T3 = 3 months post-intervention |
| Pack & Lee 2020 Korea | n = 75 Age: 51.32  TSI-HD: NR | 2 (1 intervention group, 1 control group) | To investigate the effect of a smartphone application-based dietary self-management program for haemodialysis patients and to examine its effects on biochemical indicators, self-efficacy and quality of life comparing an intervention and control group. | • Individual face-to-face • Application based | • Nurses | T1 = baseline T2 = 8 weeks post-intervention T3 = 12 weeks post-intervention |
| Ren et al. 2019 China | n = 120 Age: 44  TSI-HD NR | 2 (1 intervention group, 1 control group) | To investigate the effects of a trans theoretical model-based WeChat health education programme on self-management in hemodialysis patients comparing an intervention and control group | • Application based • Text messages | • Nurses | T0 = baseline T1 = 3 months post-intervention T2 = 21 months post-intervention |
| Griva et al. 2018 Singapore | n = 235 Age: 53.5  TSI-HD: 5.68 years (mean) | 2 (1 intervention group, 1 control group) | To investigate the long-term development of anxiety and depression in hemodialysis patients, to identify predictors of these trajectories over a 12-month period, and to evaluate the effectiveness of the Hemodialysis Self-Management Randomized Trial (HED SMART) compared to usual care on anxiety and depression symptoms. | • Group format face-to-face • Booklet • Phone calls | • Trained renal health care professionals (a medical social worker plus a renal nurse or renal dietician) | T1 = baseline T2 = 1 week post-intervention (p. i.)  T3 = 3 months p. i. T4 = 9 months p. i. |
| **Table S.2. (continued)** |  |  |  |  |  |  |
| **Study, year & origin** | **Sample size, mean age in years, time since initiation of hemodialysis (TSI-HD)** | **Group number** | **Study Aim** | **Delivery format** | **Providers** | **Data collection points** |
| Griva et al. 2017 Singapore | n = 235 Age: 53.5  TSI-HD: 5.68 years (mean) | 2 (1 intervention group, 1 control group) | To investigate the effectiveness of the HED-SMART self-management training program on the effect of adherence, self-management skills, and clinical outcomes in patients undergoing maintenance hemodialysis compared to usual care. | • Face-to-face group format • Booklet • Phone calls | • Trained renal health care professionals (a medical social worker plus a renal nurse or renal dietician) | T1 = baseline T2 = 1 week post-intervention (p. i.) T3 = 3 months p. i. T4 = 9 months p. i. |
| Liu et al. 2016 China | n = 86 Age: 43,0*  TSI-HD: 6.3 months (mean)* | 2 (1 intervention group, 1 control group) | To investigate the impact of a health education model centered on knowledge, attitudes, and behavior on the acquisition of disease-related knowledge and self-management practices among patients undergoing maintenance haemodialysis, comparing outcomes between control and intervention group. | • Group format face-to-face  • Written materials • Lectures • Telephone | • Nurses | T1 = baseline T2 = 6 months post-intervention |
| Karavetian & Ghaddar 2012 Lebanon | n = 122 Age: 58.0  TSI-HD: 5.1 years (mean) | 3 (2 intervention groups, 1 control group) | To investigate the impact of self-management dietary counseling (SMDC) on adherence to dietary guidelines for managing hyperphosphatemia in hemodialysis patients comparing across three groups: full intervention, partial intervention and control. | • Individual face-to-face • Interactive games | • Hospital dietitian | 2 measurements each week for 8 weeks |
| Moattari et al. 2012 Iran | n = 48 Age: 38.2  TSI-HD: 30.2 months (mean) | 2 (1 intervention group, 1 control group) | To investigate the effect of an empowerment program on self-efficacy, quality of life, clinical indicators of blood pressure and interdialytic weight gain and laboratory results in hemodialysis patients comparing a control and intervention group. | • Group format face-to-face  • Telephone | • Nurses | T1 = baseline T2 = 6 weeks post-intervention |
| **Table S.2. (continued)** |  |  |  |  |  |  |
| **Study, year & origin** | **Sample size, mean age in years, time since initiation of hemodialysis (TSI-HD)** | **Group number** | **Study Aim** | **Delivery format** | **Providers** | **Data collection points** |
| Lii et al. 2006 Taiwan | n = 60 Age: NR  TSI-HD:  48.9 months (mean | 2 (1 intervention group, 1 control group) | To investigate the effects of group intervention based on self-management theory on depression, self-efficacy and quality of life in haemodialysis patients comparing an intervention and control group | • Group format face-to-face | • Nurses | T1 = baseline T2 = 1 month post-intervention |
| Tsay et al. 2004 Taiwan | n = 50 Age: 51.18  TSI-HD: NR | 2 (1 intervention group, 1 control group) | To investigate the effectiveness of an empowerment program on empowerment level, self-care, self- efficacy and depression comparing an intervention and control group | • Individual face-to-face | • Nurses | T1 = baseline T2 = 6 weeks post-intervention |
| **Abbreviations:** **BDI**: Beck Depression Inventory, **CA x P**: Calcium and Phosphate product, **CDSES:** Chronic Disease Self-Efficacy Scale**, CG:** Control group, **ES:** Modified Empowerment Scale, **HADS:** Hospital Anxiety and Depression Scale, **IDWG:** Intradialytic weight gain, **IG**: Intervention group, **K10**: Kessler distress scale, **KDQOL-SF**: Kidney Disease Quality of Life Instrument-Short Form, **PDnA**: Patient dietary non-adherence**, PHQ-9**: Patient Health Questionnaire, **PK:** Patient knowledge**, PSSS**: Perceived Social Support Scale, **PSQI:** Pittsburgh Sleep Quality Index, **QoL**: Quality of Life, **RPFS:** Revised Piper Fatigue Scale, **SF-36:** The Medical Outcomes Study 36-Item Short Form, **SUPPH:** Self-care self-efficacy, **SPAN:** School Physical Activity and Nutrition, **WHO:** World Health Organization.  *****data were calculated from the available data of the study | | | | | | |
